# Improving compliance with swallowing exercise to decrease radiotherapy-related dysphagia in patients with head and neck cancer

**DOI:** 10.1016/j.apjon.2022.100169

**Published:** 2022-11-19

**Authors:** Jizhe Zhu, Xin Wang, Suxiang Chen, Ruofei Du, Haoning Zhang, Menghan Zhang, Mengwei Shao, Changying Chen, Tao Wang

**Affiliations:** a College of Nursing and Health, Zhengzhou University, Zhengzhou, China; b Centre for Molecular Medicine and Innovative Therapeutics, Health Futures Institute, Murdoch University, Murdoch, WA, Australia; c Department of Quality Control, The First Affiliated Hospital of Zhengzhou University, Zhengzhou, China; d Telethon Kids Institute, Perth, WA, Australia; e Medical School, University of Western Australia, Perth, WA, Australia; f People’ s Hospital of Hebi, Hebi, China; g Academy of Medical Sciences of Zhengzhou University, Zhengzhou, China

**Keywords:** Head and neck cancer, Dysphagia, Swallowing exercise, Compliance, Influence factor, Rehabilitation

## Abstract

**Objective:**

Dysphagia, one of the most common complications in head and neck cancer (HNC) treated with radiotherapy, can severely affect patients’ quality of life. Currently, because no “gold standard” treatment exists, swallowing exercise remains the main rehabilitation strategy for dysphagia. However, patients’ compliance with long-term swallowing exercise is only 40%, thus, greatly compromising outcomes. This article aims to analyze thefactors influencing swallowing exercise compliance in patients with HNC and explains strategies developed to date for improved rehabilitation outcomes.

**Methods:**

Research studies published from inception to 2022 were retrieved from seven databases: PubMed, Cochrane Library, Embase, CINAHL, CNKI, Wan Fang Database, and VIP Database, and 21 articles were shortlisted and systematically reviewed.

**Results:**

The swallowing exercise compliance in patients with HNC undergoing radiotherapy was affected by multiple factors, including socio-demographic factors, illness-associated factors, treatment-associated factors, and psychosocial factors. Regarding the interventions, current strategies mainly address psychosocial issues via developing various education programs.

**Conclusions:**

Different factors influencing swallowing exercise compliance are important and should be observed. Measures including developing multidisciplinary teams, applying innovative equipment, refining the intervention procedure, and applying systematic theory frameworks should be performed to achieve better outcomes of compliance interventions.

## Introduction

Head and neck cancer (HNC), the sixth most common cancer worldwide, refers to malignant tumors located from the skull base and supraclavicle to the anterior cervical spine. HNCs include neck cancer, otorhinolaryngological cancer, and oral and maxillofacial cancer.^1^^,^^2^ The Global Burden of Disease study has estimated that 890,000 new HNCs occurred worldwide in 2017 and has indicated an increased incidence rate in recent years.^1^ Currently, radiotherapy is the most important treatment option for patients with HNC, approximately 80% of whom receive radiotherapy at least once.^3^ However, whereas tumor cells are irradiated during radiotherapy, normal cells adjacent to the tumor are also exposed, thereby resulting in a series of adverse effects, ranging from relatively mild tissue fibrosis, reduced saliva secretion, or local tissue swelling to life-threatening dysphagia.^3^ Indeed, dysphagia is considered a major concern for patients with HNC undergoing radiotherapy^4^ because of the local pressure caused by the tumor mass effect or the invasion of tumors into swallowing-associated tissues.^5^ After radiotherapy, 72.4% of patients with HNC have been reported to develop solid food dysphagia, and 17.2% develop liquid dysphagia.^4^

Dysphagia can cause sensory changes (such as taste changes or mucosal pain), which results in appetite loss or eating difficulty, malnutrition, and dehydration.^4^^,^^6^ Moreover, patients with dysphagia tend to avoid attending social activities and to have psychological disorders (such as depression and cognitive impairment).^7^ Moreover, the patients’ lives can be threatened if dysphagia-associated aspiration pneumonia develops.^8^

Currently, because no “gold standard” treatment can alleviate the damage caused by radiotherapy-associated dysphagia, different types of swallowing exercises have been introduced and confirmed to be beneficial for patients with HNC.^9^ Swallowing exercises include active exercise and passive exercise. Active exercises include bulging the cheek or mouth, knocking the teeth, performing swallowing actions, stretching the tongue, and rolling the tongue. Passive exercises mainly include actions around the throat, tongue, and jaw, such as Masako maneuvers and Mendelsohn swallowing.^10^^,^^11^ Generally, a typical swallowing exercise includes 4 to 5 actions depending on patients’ individual needs. The importance of adhering to swallowing exercises has been well documented. As shown in a study led by Starmer, compared with noncompliant patients, patients with better compliance with an exercise program show better tolerance to regular diet (54.4% vs. 21.4%), a lower G-tube dependence (22.8% vs. 53.6%), and a higher rate of adhering to a self-managed diet (54.4% vs. 25.0%).^12^ Another study has found that patients with > 50% swallowing exercise compliance, compared with < 50% compliance, score much higher in swallowing-associated quality of life.^13^

However, despite the effectiveness of swallowing exercises, a lack of compliance is common. The long-term exercise compliance of patients with HNC is only 40%,^14^ thus inevitably affecting rehabilitation outcomes. Here, through an integrative review, we explored the factors influencing swallowing exercise compliance in patients with HNC, then analyzed the advantages and disadvantages of existing intervention strategies to address this issue. The ultimate goal was to help health care providers develop effective interventions to improve the outcomes of patients with HNC with radiotherapy-associated dysphagia.

## Methods

This is an integrative review study on the challenges of improving compliance with swallowing exercises in patients with HNC. This integrated review of literature summarized previous studies by extracting the study results according to the Russell model, which comprises 5 steps: (1) problem identification; (2) literature search; (3) evaluation of data; (4) data analysis; and (5) interpretation and presentation of the results.^15^

### Problem identification

This study was planned to explore the factors influencing swallowing exercise compliance in patients with HNC and to determine the advantages/disadvantages of existing intervention strategies and corresponding solutions. Three key questions were used to guide the review process: (1) What factors influence swallowing exercise compliance in patients with HNC? (2) How should the compliance of swallowing exercise be evaluated? (3) How can compliance of swallowing exercise in patients with HNC be improved? Answering these key questions will help health care providers develop effective interventions to improve the outcomes of patients with HNC with radiotherapy-associated dysphagia.

### Literature search

The following databases were searched from inception to August 2022: PubMed, Cochrane Library, Embase, CINAHL, CNKI, Wan Fang Database, and VIP Database. The reference lists of relevant articles were also searched. The following Medical Subject Headings were used in the search: “head and neck neoplasms,” “cancer of head and neck,” “exercise therapy,” “rehabilitation," “self-management,” “swallow training,” “swallowing exercise,” “deglutition training,” “rehabilitation program,” “rehabilitation plan,” “rehabilitation intervention,” “rehabilitative intervention,” “rehabilitative interventions,” “patient compliance,” “adherence,” “compliance,” “patient adherence,” “patient non-compliance,” “implementation rates,” and “execution rate.” For detailed search strategies, please see the “Appendix.”

### Data evaluation

The inclusion criteria in this study were developed on the basis of the analysis of participants, treatments, and outcomes. The detailed inclusion criteria were as follows: (1) patients with HNC with tumors in the oral cavity, oropharynx, nasopharynx, larynx, hypopharynx, parotic gland, and parotic gland; (2) patients aged ≥ 18 years; (3) patients planning radiotherapy or who underwent radiotherapy; (4) outcomes including compliance with preventive swallowing exercises or swallowing exercises after radiotherapy; and (5) articles written in either Chinese or English. The exclusion criteria were (1) articles for which the original article and detailed methods could not be accessed and (2) articles irrelevant to the research questions.

### Data analysis

**Fig. 1:**
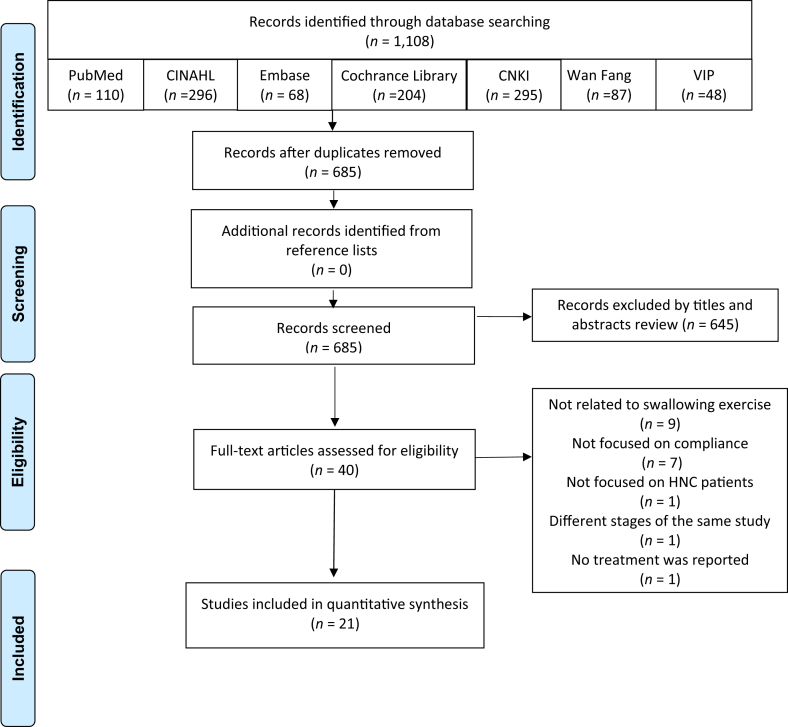
A schematic of the sampling process.

Two reviewers independently screened the title and abstract of each article to assess its relevance before reviewing the full texts of potential studies. As shown in Fig. 1, a total of 1108 articles were identified from databases. After the removal of duplicates, 685 remaining articles were screened for relevance. Eventually, 40 articles were selected for full-text checking, and 21 articles met the inclusion criteria and were included in this integrative review.

Next, data including the first author, year of publication, study design, participants, setting, intervention, comparison, and outcome variables were extracted and imported into Note Express. The extracted data were cross-checked for accuracy.

## Results

### Study design and patient characteristics

In terms of study design, the 21 selected studies (Table 1)^10^^,^^13^^,^^14^^,^^16^, ^17^, ^18^, ^19^, ^20^, ^21^, ^22^, ^23^, ^24^, ^25^, ^26^, ^27^, ^28^, ^29^, ^30^, ^31^, ^32^, ^33^ comprised 6 randomized controlled studies,^10^^,^^17^^,^^18^^,^^27^^,^^29^^,^^32^ 8 quasi-experimental studies,^13^^,^^16^^,^^19^, ^20^, ^21^^,^^23^^,^^25^^,^^28^ 6 observational studies.^14^^,^^24^, ^25^, ^26^^,^^31^^,^^33^ and 1 qualitative study.^22^ Regarding the radiotherapy stage, 7 studies focused on patients planning radiotherapy,^10^^,^^13^^,^^14^^,^^19^^,^^20^^,^^22^^,^^30^ 12 studies focused on the patients completed radiotherapy,^16^^,^^18^^,^^21^, ^22^, ^23^, ^24^, ^25^^,^^27^, ^28^, ^28^, ^29^^,^^31^^,^^32^ and only 1 study focused on patients in pre-radiotherapy, mid-radiotherapy, and post-radiotherapy.^33^ The typical swallowing exercise included: effortful swallow,^13^^,^^16^^,^^18^, ^19^, ^20^, ^21^ the Mendelsohn maneuver, the Masako maneuver,^13^^,^^16^^,^^19^^,^^20^ and mouth-opening exercise.^19^^,^^24^, ^25^, ^26^, ^27^, ^28^, ^29^, ^30^, ^31^, ^32^, ^33^ Furthermore, Jaw stretches,^13^^,^^19^^,^^23^ tongues stretching and strengthening,^18^^,^^19^^,^^29^^,^^32^ tapping teeth, chewing, and blowing^24^^,^^29^^,^^32^ were also reported.

**Table 1:** Study and patient characteristics (*n* ​= ​21).

First author, year, language	Study design	Sample size (I/C)	Participant characteristics	Swallow training regimen
Starmer H, 2022, English	Quasi-experimental study	44/47	Cancer type: HNC (oral cavity, oropharynx, nasopharynx, larynx, and hypopharynx)	During radiation
			Age (years): 59.3 ​± ​9.9/61.0 ​± ​9.6	
			Gender: male (85.7%/87.2%)	
			Treatment: radiation	
			Timepoint: planning radiation	
			Dysphagia stage: NR	
Constantinescu G, 2021, English	Quasi-experimental study	20	Cancer type: HNC (oral, oropharyngeal, or other cancers)	After radiation A target of 72 swallows per day split between 3 different exercise types: 3 regular swallows, 3 effortful swallows, and 3 Mendelsohn maneuver swallows
			Age (years): 61 ​± ​8	
			Gender: male (75%)	
			Treatment: surgery ​± ​(chemo)radiation	
			Time point: 3 months post-HNC treatment	
			Dysphagia stage: may benefit from the Mendelsohn maneuver swallow(depend on SLP)	
Hajdú SF, 2019, English	Quasi-experimental study	45	Cancer type: HNC (oral cavity, larynx, oropharynx, hypopharynx, or unknown primary tumor) Age (years): 62 (41–78) Gender: male (78%) Treatment: (chemo)radiotherapy Timepoint: planning radiation Dysphagia stage: NR	During radiation
				Three times daily
				14 exercises (tongue stretching and strengthening, jaw mobility and mouth opening, Mendelsohn maneuver, Shaker exercise, Masako maneuver, Effortful swallow, and Valsalva)
Starmer HM, 2018, English	Quasi-experimental study	36	Cancer type: HNC (oropharyngeal tumors 83%)	During radiation 3 sets of 10 reps of each exercise twice daily Specific exercises included effortful swallow, Masako, Mendelsohn, effortful pitch glides, and jaw stretches
			Age (years): 61 ​± ​8	
			Gender: male (80%)	
			Treatment: Chemoradiation (75%) Surgery ​+ ​radiation (17%) Radiation (8%)	
			Timepoint: planning radiation	
			Dysphagia stage: NR	
Cnossen IC, 2017, English	Quasi-experimental study	50	Cancer type: HNC (oral cavity, oropharynx, hypopharynx, or larynx)	After radiation At least once a day for 15 ​min and preferably 3 times a day Swallowing with strength: effortful swallow, taking sips of water regularly
			Age (years): 66 (40–77)	
			Gender: male (78%)	
			Treatment: (chemo)radiotherapy	
			Time point: terminate radiotherapy	
			Dysphagia stage: RTOG scores: 2-4	
Wall LR, 2017, English	Randomized controlled study	20/25/26	Cancer type: HNC (100%)	During radiation Usual swallowing rehabilitation based on a literature review
			Age (years): 58 ​± ​8	
			Gender: male (89%)	
			Treatment: (chemo)radiotherapy	
			Time point: planning radiation	
			Dysphagia stage: ​FOIS: 7/≤ 6	
Shinn EH, 2013, English	Cohort	109	Cancer type: oropharyngeal cancer (100%)	Preradiotherapy/mid-radiotherapy/postradiotherapy Speech pathology-led swallowing exercises
			Age (years): 57 ​± ​9	
			Gender: male (87%)	
			Treatment: (chemo)radiotherapy	
			Time point: planning radiation	
			Dysphagia stage: NR	
Govender R, 2020, English	Randomized controlled study	16/16	Cancer type: HNC (oral cavity, nasopharynx, Oropharynx, hypopharynx, larynx)	After radiation Usual posttreatment swallowing rehabilitation
			Age (years): 58.56 ​± ​12.41/55.19 ​± ​9.45	
			Gender: male (95%)	
			Treatment: radiotherapy ​± ​surgery (2%) ​± ​chemotherapy	
			Time point: planning radiation (radiotherapy group)	
			Dysphagia stage: NR	
Govender R, 2017, English	Qualitative	13	Cancer type: HNC (oral cavity, nasopharynx, oropharynx, hypopharynx/larynx)	After radiation Depend on swallowing exercise consulting with a SLT
			Age (years): 56.5 ​± ​6.5	
			Gender: male (69%)	
			Treatment: radiotherapy ​± ​surgery ​± ​chemotherapy	
			Time point: terminate radiotherapy	
			Dysphagia stage: PSS: < 50: 4 PSS: ≥ 50: 9	
Kraaijenga SA, 2017, English	Quasi-experimental study	18	Cancer type: HNC (parotic gland, parotic gland, hypopharynx, oral cavity, neck metastasis, oropharynx,larynx)	After radiation Perform the SEA exercises 3 times daily for at least 6 weeks and for a maximum of 8 ​weeks Progressive muscle overload, including chin tuck, jaw opening, and effortful swallow exercises
			Age (years): 65 (42–74)	
			Gender: male (76%)	
			Treatment: (chemo)radiotherapy	
			Time point: The dysphagia had to be persistent for at least 1 year	
			Dysphagia stage: PAS ≤ 4	
Baudelet M, 2020, English	Randomized controlled study (protocol)	50/50/50	Cancer type: HNC (100%)	Before and during radiation 5 times/wk (30–40 ​min) Tongue strengthening exercises and chin tuck against resistance combined with an effortful swallow
			Treatment: (chemo)radiotherapy	
			Time point: 4 weeks before (chemo)radiotherapy	
Wen K, 2021, Chinese	Cross-sectional study	124	Cancer type: NPC (100%)	After radiation Mouth opening exercise
			Age (years): ＜ 60:78 ​≥ ​60:46	
			Gender: male (66%)	
			Treatment: radiotherapy	
			Time point: terminate radiotherapy	
			Dysphagia stage: NR	
Zhang YF, 2020, Chinese	Cross-sectional study	88	Cancer type: NPC (100%)	After radiation (1) Mouth opening exercise lasting 5 ​s, more than 60 times a day; (2) Tapping teeth, 100 times/time, 3 times/d; (3) Drum gills, chewing gum, blowing balloons, and other exercises; (4) Neck exercise 3–5 ​min/time, 5 times/d
			Age (years): 35–60	
			Gender: male (49%)	
			Treatment: radiotherapy	
			Time point: terminate radiotherapy	
			Dysphagia stage: NR	
Xu X, 2018, Chinese	Quasi-experimental study	118/121	Cancer type: NPC (100%)	After radiation Mouth opening exercise
			Age (years): 25–65	
			Gender: male (76.03%)	
			Treatment: radiotherapy	
			Time point: terminate radiotherapy	
			Dysphagia stage: NR	
He PY, 2015, Chinese	Randomized controlled study	143/144	Cancer type: NPC (100%)	After radiation (1) Mouth opening exercise, 10–15 ​min/time, 3 times/d; (2) Tapping teeth, 100 times/time, 3 times/d; (3) Tongue exercise, 10 times/d, each interval of 10 min; (4) Drum cheeks, chewing, whistling, deep breathing exercise, 10–15 ​min/time, 3 times/d; (5) Temporomandibular joint massaging, 10–15 ​min/time, 3 times/d
			Age(years): 47 ​± ​11	
			Gender: male (76.1%)	
			Treatment: radiotherapy	
			Time point: terminate radiotherapy	
			Dysphagia stage: NR	
Yu JF, 2021, Chinese	Randomized controlled study	40/40	Cancer type: NPC (100%)	After radiation Mouth opening exercise
			Age (years): 40 ​± ​6	
			Gender: male (68.8%)	
			Treatment: radiotherapy	
			Time point: terminate radiotherapy	
			Dysphagia stage: NR	
Fan SQ, 2021, Chinese	Cross-sectional study	150	Cancer type: NPC (100%)	During radiation and after radiation Mouth opening exercise
			Age (years): 40 ​± ​6	
			Gender: male (68.8%)	
			Treatment: radiotherapy	
			Time point: undergoing radiotherapy for more than 4 weeks	
			Dysphagia stage: NR	
Wang XM, 2017, Chinese	Quasi-experimental study	84	Cancer type: NPC (100%)	During radiation Mouth opening exercise
			Age (years): 17–72	
			Gender: male (70.2%)	
			Treatment: radiotherapy	
			Time point: planning radiotherapy	
			Dysphagia stage: NR	
Lu XN, 2017, Chinese	Randomized controlled study	60/60	Cancer type: NPC (100%)	After radiation Mouth opening exercise, drum gill, shrink gill exercise, tongue exercise, knock teeth exercise, temporomandibular joint exercise, neck muscle exercise
			Age (years): 24–69	
			Gender: male (78%)	
			Treatment: radiotherapy	
			Time point: terminate radiotherapy	
			Dysphagia stage: NR	
Chen PJ, 2016, Chinese	Cross-sectional study	124	Cancer type: NPC (100%)	After radiation Mouth opening exercise
			Age (years): < 60:78 ​≥ ​60:46	
			Gender: male (66.1%)	
			Treatment: (chemo)radiotherapy	
			Time point: terminate radiotherapy	
			Dysphagia stage: NR	
You GM, 2005, Chinese	Cross-sectional study	50	Cancer type: NPC (100%)	Preradiotherapy/midradiotherapy/postradiotherapy Mouth opening exercise: mouth opening to the maximum and then slowly closed, repeated 10 rounds, 3 times a day; bite cork 10–15 min, 1 times a day
			Age (years): ＜ 60:78 ​≥ ​60:46	
			Gender: male (68%)	
			Treatment: radiotherapy	
			Time point: planning radiotherapy	
			Dysphagia stage: NR	

FOIS, Functional Oral Intake Scale; HNC, head and neck cancer; I/C, intervention/control; IOPI, Iowa Oral Performance Instrument; NPC, nasopharyngeal carcinoma cancer; NR, not report; PAS, penetration-aspiration scale; PSS, Performance Status Scale; RTOG, Radiation Therapy Oncology Group; SEA, Swallow Exercise Aid; SLPs, speech and language pathologist; SLTs, speech and language therapists.

### Compliance evaluation

#### Evaluation methods

The evaluation methods were performed either subjectively or objectively, and the implementation included compliance quantification^13^^,^^14^^,^^16^^,^^18^^,^^20^^,^^23^, ^24^, ^25^, ^26^^,^^28^^,^^31^^,^^32^ and patient self-report.^10^^,^^17^, ^18^, ^19^^,^^21^^,^^23^^,^^25^^,^^26^^,^^28^^,^^31^^,^^32^ As presented in Table 2, subjective evaluation was used in most (17/22) studies including observation,^14^^,^^18^^,^^27^^,^^29^^,^^30^^,^^33^ inquiry,^10^^,^^18^^,^^21^ questionnaire surveys,^17^^,^^23^, ^24^, ^25^, ^26^^,^^28^^,^^32^ and face-to-face interviews.^22^ In contrast, only 2 studies used novel equipment for evaluating compliance objectively.^16^^,^^18^ For example, a mobile health (m-Health) system, Mobili-T, was used to collect the number of swallowing exercises on the basis of surface electromyography data for swallowing muscles.^16^ In another case, a device called the Iowa Oral Performance Instrument, which was equipped with a digital display function and was connected to an air-filled bulb, was used. After the bulb was placed into the patient's mouth, the patient was instructed to push the bulb as forcefully as possible against the palate to perform tongue strengthening exercises, and the number of exercises performed was recorded automatically. Furthermore, the degree of compliance was assessed on the basis of the time spent using these exercise training devices/apps.^18^ One study was a mixed subjective and objective evaluation.^18^

**Table 2:** Evaluation methods of compliance (*n* ​= ​20).

First author and year	The tool used to measure compliance	Definition of compliance	Compliance measuring time	Duration
Starmer H, 2022	Logs of an m-Health app: HNC virtual coach and paper logs	Percent trials completed of trials prescribed Input	Once a week during radiotherapy	7 weeks
Constantinescu G, 2021	An m-Health system: Mobili-T	Percent trials completed of trials prescribed	Once a week during the intervention	6 weeks
Hajdú SF, 2019	Participants' training logs	Percentage of prescribed exercises completed	Once a week during radiotherapy	7 weeks
Starmer HM, 2018	Logs of the Vibrant mobile application	Percentage of prescribed logs completed	Per exercise	7 weeks
Cnossen IC, 2017	Patients' diaries on paper or online	Percentage of patients who kept up exercising and exercise performance level	T1: 6th weeks during the intervention	12 weeks
			T2: 12th weeks during the intervention	
Wall LR, 2017	Exercise log books and an m-Health system: Swallow-IT	Percentage of prescribed exercise completed	Once a day	6 weeks
Shinn EH, 2013	Speech pathologists' documentation	Demonstrated adequate competency in all assigned swallowing exercises to a speech pathologist or not	T1: ​weeks 3–6 during radiation	2 years
			T2: Six months after completion of radiation	
			T3: 1–2 years after completing radiation	
Govender R, 2020	A study questionnaire	Percentage of patients with satisfactory to good adherence based on the responses to the adherence form	T1: 1 month during the intervention	6 months
			T2: 3rd month during the intervention	
			T3: 6th month during the intervention	
Kraaijenga SA, 2017	A study-specific questionnaire	NR	6–8 weeks after intervention	∖
Baudelet M, 2020	Paper registration of patients and therapists and an equipment: IOPI	The total number of exercises performed per week and the time spent on the app	During the first 4 weeks of radiotherapy	4 weeks
Wen K, 2021	A self-designed questionnaire	Depend on the 3 factors: compliance with mouth opening exercise, compliance with precautions, and compliance with advice-seeking	3 months after completion of radiotherapy	∖
Zhang YF, 2020	A self-designed compliance questionnaire	Depending on patients' scores in different exercise actions:1 point means no exercise, and 2 points mean sometimes exercise	NR	∖
Xu X, 2018	A compliance questionnaire	Depending on the 3 factors: faith, will, and confidence for mouth opening exercise	Six months after radiotherapy	∖
He PY, 2015	A compliance questionnaire	Complete compliance: fully completed the prescribed actions	After radiotherapy and 3 months after discharge	3 months
		Partial compliance: partially completed prescribed actions		
		Noncompliance: occasionally exercised or never exercise		
Yu JF, 2021	Nurse(s)	Complete compliance: fully completed the prescribed actions	After 6 months of intervention	∖
		Partial compliance: partially completed prescribed actions		
		Noncompliance: occasionally exercised or never exercise		
Fan SQ, 2021	Mouth opening exercise compliance questionnaire	Depend on the 3 factors: compliance with mouth opening exercise, compliance with precautions, and compliance with advice-seeking	The first review at the end of radiotherapy	∖
Wang XM, 2017	Nurse(s)	Complete compliance: fully completed the prescribed actions	Terminate radiotherapy	∖
		Partial compliance: partially completed prescribed actions		
		Noncompliance: occasionally exercised or never exercise		
Lu XN, 2017	Nurse(s)	Full compliance: fully completed the prescribed actions	12 months after discharge	∖
		Partial compliance: partially completed prescribed actions; including the action is not in place, the number of training is not enough;		
		Noncompliance: never exercise or mouth opening exercise less than 10 times a day		
Chen PJ, 2016	A self-designed compliance questionnaire	Depend on the compliance of seeking advice and the compliance of precautions	3 months after radiotherapy	∖
You GM, 2005	Nurse(s)	The degree of consistency between patient ' exercise actions and prescribed action	T1: Preradiotherapy	During radiotherapy
			T2: Midradiotherapy	
			T3: Postradiotherapy	

HNC, head and neck cancer; IOPI, Iowa Oral Performance Instrument; NR, not report; SLPs, speech and language pathologist.

#### Compliance level

Compliance levels were classified with 2 methods: (1) a continuous method, in which compliance was divided into 3 or more levels (full compliance/partial compliance/noncompliance)^14^^,^^18^^,^^24^, ^25^, ^26^, ^27^, ^28^, ^29^, ^30^, ^31^, ^32^ and (2) a dichotomous method, in which compliance was divided into 2 levels (high compliance or low compliance)^19^ or was represented as a percentage determined by the researchers.^10^^,^^13^^,^^16^^,^^17^^,^^19^, ^20^, ^21^ Three studies evaluated patient compliance on the basis of compliance-associated factors, including taking precautions, help-seeking, faith, willingness, self-efficacy, and the ability to complete the assigned swallowing exercises.^14^^,^^25^^,^^28^

#### Time points

The range of measurement baseline spanned the first day of radiotherapy^10^^,^^20^ to 12 months after radiotherapy.^29^ The duration of evaluation ranged from 6 weeks^10^^,^^17^ to 2 years,^14^ and the most common evaluation frequency was once per week.^13^^,^^16^^,^^19^

### Factors influencing compliance

Thirteen studies reported factors influencing swallowing exercise compliance,^10^^,^^13^^,^^14^^,^^16^^,^^19^^,^^21^^,^^22^^,^^24^, ^25^, ^26^^,^^30^^,^^31^^,^^33^ as detailed below.

#### Sociodemographic factors

Six studies showed that male sex, older age, smoking, low education level, and residence in rural areas were negative factors influencing patients' compliance with swallowing exercises.^10^^,^^24^, ^25^, ^26^^,^^31^^,^^33^ Interestingly, 2 studies indicated that sex and age had no significant effects on compliance with swallowing exercises.^19^^,^^24^ Moreover, economic factors, such as income and the method of paying for the treatment, affected compliance with swallowing exercises.^25^^,^^31^ For example, as shown in a study led by Chen, patients with nasopharyngeal cancer whose per capita monthly household income exceeded 3000 Chinese yuan showed better compliance with mouth opening exercises than patients with lower incomes, and the compliance rate increased gradually with increasing income.^31^ In addition, according to Wen's study, the method of paying for the treatment, such as the self-paid medical care model, medical insurance model, or government-paid medical care model, was also a significant factor influencing patient compliance with swallowing exercises.^25^

#### Illness-associated factors

Although patients with oropharyngeal tumors have been reported to be less likely to complete exercises than patients with tumor sites in the oral cavity, oropharynx, nasopharynx, larynx, or hypopharynx,^13^ as shown in a study led by Hajdú, no significant relationship between swallowing exercise compliance and tumor site (oral cavity, oropharynx, larynx, or hypopharynx) was found.^19^ According to the selected studies, the tumor differentiation grade,^31^ HNC duration,^13^^,^^24^ and complications^10^ affected swallowing exercise compliance. For example, a higher tumor differentiation grade was associated with lower compliance scores,^31^ and patients with an HNC duration > 1 year had poor compliance with exercise during radiotherapy.^24^ In another study, after 6 weeks of exercise, patients 2–5 years postradiation demonstrated higher adherence than patients < 2 years posttreatment.^16^

#### Treatment-related factors

The treatment-associated factors of radiotherapy course,^13^^,^^33^ radiation-associated adverse effects (such as pain, fatigue, and nausea),^13^^,^^14^^,^^30^ and involvement of concomitant therapy (chemotherapy) significantly affected compliance with swallowing exercises.^10^^,^^21^ Two studies reported that compliance with swallowing exercises gradually decreased after radiotherapy.^13^^,^^33^ Moreover, the pain of radiation stomatitis, fatigue, or nausea sometimes even resulted in suspension of functional exercises.^30^ In addition, compared with patients treated with radiotherapy only, patients receiving concomitant chemotherapy had lower exercise frequency.^10^^,^^21^

#### Psychosocial factors

##### Social support

Most (13) of the assessed studies reported that better social support increased swallowing exercise compliance.^10^^,^^13^^,^^16^^,^^17^^,^^19^, ^20^, ^21^, ^22^^,^^24^^,^^28^, ^29^, ^30^^,^^32^ For example, in a study led by Wall, patients with HNC who underwent self-exercise, compared with clinician-directed face-to-face therapy and m-Health application-assisted therapy, showed the lowest adherence to swallowing exercises.^10^ Generally, the forms of social support included notification reminders, educational videos, remote monitoring based on m-Health systems,^10^^,^^13^^,^^16^^,^^18^^,^^20^ face-to-face instruction sessions,^10^^,^^16^^,^^18^^,^^19^^,^^21^^,^^22^ and peer education.^28^

##### Cognition and belief

According to our analysis, in most cases, the lack of compliance with swallowing exercises was due to a lack of understanding regarding their importance or to insufficient motivation.^10^^,^^14^^,^^16^^,^^22^^,^^24^, ^25^, ^26^^,^^31^ Three studies reported that self-management efficacy, which was associated with attitudes toward treatment and self-decision-making, was positively correlated with compliance with swallowing exercises.^25^^,^^26^^,^^31^

### Characteristics of the interventions

#### Deliverer

Among all included studies, the interventions were delivered by different personnel, including speech and language pathologists (SLPs),^13^^,^^16^, ^17^, ^18^^,^^20^^,^^21^ speech therapists,^10^^,^^19^^,^^21^ oncology specialist nurses,^27^, ^28^, ^29^, ^30^^,^^32^ oncologists,^10^^,^^28^ radiation therapists,^18^^,^^28^ and clinician researchers,^16^^,^^23^ as shown in Table 3. Five studies used multidisciplinary team-led interventions.^10^^,^^16^^,^^18^^,^^21^^,^^28^ In addition to health care providers, 1 study developed a peer education program by inviting patients with nasopharyngeal cancer postradiotherapy to perform a swallowing training program and then participate in an experience sharing session.^28^

**Table 3:** Characteristics of the interventions in studies included in the review (*n* ​= ​14).

First author and Year	Delivered by	Medium	Intervention	Control	Results that are relevant for the review
Starmer H, 2022	SLP(s)	HNC Virtual Coach app	A notification reminder and a link to a training video through the “HNC Virtual Coach” app twice a day	Did not have access to educational videos but were provided handouts before the start of RT	The adherence of patients in the app arm is higher than in the paper arm
Constantinescu G, 2021	SLP(s) and clinician researchers	An m-Health system: Mobili—T	(1) Remotely monitored exercise data and answer questions; (2) Check-in appointment with this SLP.	NR	Adherence to the exercise regimen remained high from 84% in Week 1, to 72% in Week 6
Hajdú SF, 2019	OT	OT-led swallowing exercises	(1) Designed targeted exercise programs based on the clinical examination of patients' (2) Supervised individual training and provided sessions	NR	The total cohort median adherence to exercises was 78%
Starmer HM, 2018	SLP(s)	Vibrant mobile app	(1) The application provided training videos, reminders, exercise logging, and an educational program (2) Communication between patients and providers was possible through a messaging system	NR	On average, there was a 29% adherence rate. 25% of participants logged at least 2 exercise sessions per day over the 7-week treatment period and 53% recorded at least one session per day
Cnossen IC, 2017	SLP(s) and swallowing therapist(s) and speech therapist(s)	A guided home-based prophylactic	(1) Online training including photo and video examples of the exercises, and educational booklet; (2) Face-to-face instruction session; (3) Each patient is contacted by phone in a weekly 10-min coaching session by an experienced speech therapist.	NR	The adherence rate at 6 ​weeks was 70% and decreased to 38% at 12 ​weeks.
Wall LR, 2017	Clinician and speech pathologist	(1) Clinician-directed therapy (2) Technology-assisted therapy (3) Independent patient-directed therapy	(1) Face-to-face education session by clinician; (2) The Swallow-IT application: tracks the number of repetitions and cycles completed and records patient perceptions of perceived effort when completing each exercise.	Joint speech pathology/dietetic sessions weekly	The lowest adherence was observed in the patient-directed therapy group. There was a trend for higher adherence in the Swallow-IT group.
Govender R, 2020	SLP(s)	Pretreatment swallowing intervention package: SIP SMART	(1) Patients underwent an x-ray swallow assessment that enabled a physiological analysis of swallowing and the selection of specific and targeted exercises. (2) Showed a video animation of swallowing. (3) Goal setting, self-monitoring, and behavioral practice were actively employed.	(1) One 45-min session; (2) Clinical baseline screening of swallowing and communication; (3) Information provision.	Patient-reported adherence in The intervention group is higher than the control group.
Kraaijenga SA, 2017	Clinical investigator	SEA-based exercise regimen	Exercise by an innovative tool	NR	The median compliance in terms of adherence to the 3 daily exercise sessions was 97% (range: 86%–100%).
Baudelet M, 2020	Radiation therapists and SLPs	(1) Technology-assisted exercise (2) Therapist-led exercise (3) Patients self-exercise	(1) App group: practices at home but receives continuous counseling and gets instructions by videos via an application on a tablet (2) Therapist group: was given face-to-face therapy and be counseled by an SLP 5 times per week	Perform the exercises at home, without the supervision of an SLP but with a counseling session of 10 ​min every week	NR
Xu X, 2018	Oncologist, oncology specialist nurse, radiotherapy technician, psychological consultant, and nutritionist, statistician	Peer education program	(1) Peer educators use text, pictures, videos to carry out education, and emphasize the importance of mouth exercise through online and offline communication, case sharing, and other forms (2) Organized patients to do exercise, and provided supervision	(1) Symptom prevention, control, and management; (2) Recorded the mouth opening exercise by video; (3) Encouraged and reminded patients to exercise;	The intervention group has higher training compliance than the control group.
He PY, 2015	Nurses	PDCA cycle education model	(1) Nurses checked patient's completion every day; (2) Solve problems of patients; (3) A seminar was held every 2 weeks; (4) Patients with poor compliance were guided by the nursing team leader to strengthen the patient's health knowledge and behavior.	Mouth opening exercise training, the content of functional exercise is the same as the intervention group	The intervention group has higher training compliance than the control group
Yu JF, 2021	Nurses	Pender health promotion model	Intervention based on past behavioral factors, personal factors, cognitive factors, and behavioral emotional factors.	Provided disease-related knowledge, diet and life guidance, and help patients complete functional training	The intervention group has higher training compliance than the control group.
Wang XM, 2017	Nurses	Comprehensive nursing intervention model	(1) Improve nurses' awareness of scientific research; (2) Implement health education for patient and their family members; (3) Pain intervention; (4) Communication by network platform.	NR	Patients could complete their exercises under the guidance of nurses while the willingness to complete them independently was poor.
Lu XN, 2017	Nurses	Orem self-care model	(1) Provided information about the disease and guidance to help them in self-care; (2) Theoretical knowledge, case introduction, graphic examples; (3) Strengthen communication with patients and their families and help mobilize patient's exercise initiative; (4) Encourage family members to give patients more support; (5) Encourage patients to exercise based on their abilities.	(1) Health education and exercise guidance during hospitalization; (2) Routine follow-up by a dedicated disease manager	The intervention group has higher exercise compliance than the control group

HNC, head and neck cancer; NR, not report; OT, occupational therapist; SEA, swallow exercise aid; SLP, speech and language pathologist.

#### Procedure of intervention

Most studies included the following procedures: (1) screening patients with HNC treated with radiotherapy (preradiation, during radiation, or postradiation); (2) evaluating patients’ demographic information, cancer status, and treatment information; (3) training patients and providing a swallowing exercise plan; and (4) supervising patients and encouraging them to implement their individualized exercise plans.^10^^,^^13^^,^^16^, ^17^, ^18^, ^19^, ^20^, ^21^^,^^23^^,^^27^, ^28^, ^29^, ^30^ In a study led by He, the PDCA (Plan-Do-Check-Act) cycle model was proposed to standardize education procedures. This model included the following steps: (1) plan: formulate the overall plan on the basis of literature review, questionnaires, and expert interview; (2) do: perform training in the following order: (a) emphasize the adverse effects of radiotherapy, (b) emphasize the importance of swallowing exercise, and (c) formulate an individual exercise plan; and (3) check and act: verify patient compliance and analyze existing problems to determine their causes and revise exercise plans. The PDCA training was repeated after each round until patients were discharged.^32^

#### Interventions designed to address various psychosocial factors

Before exercise, the following interventions had been used in the assessed studies: face-to-face instruction sessions,^10^^,^^13^^,^^16^, ^17^, ^18^, ^19^, ^20^, ^21^^,^^27^, ^28^, ^29^, ^30^^,^^32^ peer experience sharing,^28^ and providing training materials such as photographic examples of the exercise,^21^ training videos,^13^^,^^17^^,^^18^^,^^20^^,^^21^^,^^28^ and educational booklets including information on the importance of exercise, adverse reactions to radiotherapy, and exercise plans.^21^ During exercise, beyond refreshing patients’ memory regarding previously performed training, a series of follow-up activities were implemented by health care providers.^10^^,^^13^^,^^16^, ^17^, ^18^, ^19^, ^20^, ^21^ For example, professional appointments were implemented in person, by phone, or through e-mail to determine whether participants required additional supplies.^16^ In addition, in 1 study, an educational program focused on improving understanding among nurses and family members of patients was used to better manage patients’ swallowing exercises.^30^

#### Innovative equipment-based intervention

The recent developments of novel m-Health systems, such as the HNC Virtual Coach,^13^ Mobili-T,^16^ Vibrant Mobile,^20^ Swallow-IT apps,^10^ have brought new hope for improving compliance with swallowing exercises, through providing educational videos, enabling interactive feedback, automatically recording exercise data, and providing notification reminders. In addition, Kraaijenga developed a physical tool called “The Swallow Exercise Aid” based on an existing swallowing exercise tool, which featured a feedback device to prompt patients to progressively add exercise load by increasing exercise resistance.^23^

#### Theoretical framework underlying the intervention

According to our analysis, 14 of the 21 studies^10^^,^^13^^,^^14^^,^^16^^,^^17^^,^^19^, ^20^, ^21^^,^^23^^,^^27^, ^28^, ^29^, ^30^^,^^32^ took measures to improve swallowing exercise compliance in patients with HNC on the basis of behavior change strategies (BCTs), such as goal setting, self-monitoring, and prompting.^34^ Pender's Health Promotion Model helped health care providers provide targeted swallowing interventions based on patients' behavioral factors, demographic factors, cognitive factors, and emotional factors.^35^ For inpatients, Orem self-care theory was used to formulate mouth opening exercise plans on the basis of patients' self-care agency relating to consciousness, vital signs, and exercise tolerance, whereas for outpatients, the supportive-educative system was formally used.^29^

## Discussion

### Combined subjective and objective evaluation enables more reliable compliance assessment

Currently, widely accepted methods or tools for evaluating swallowing exercise compliance in patients with HNC are lacking.^36^ As a result, the evaluation is highly dependent on how researchers define the concept of compliance. To evaluate the swallowing exercise compliance of patients with HNC, different researchers may have different standards. For example, Starmer has used the percentage of completed trials as an indicator of patients' compliance with swallowing exercise and used an m-Health application to record the times of exercise automatically.^13^ However, in another study, although an m-Health system was also used to record the times of exercise, the total number of exercises performed per week and the time spent on the app were used to indicate patients’ compliance with swallowing exercises.^18^ In addition, as shown in a study led by Shinn, compliance with long-term exercise (from the initial radiation to 1–2 years after radiotherapy) depended on whether patients were able to fully complete swallowing exercises at the following specific time points: (1) 3–6 weeks during radiation, (2) 6 months after completion of radiation, and (3) 1–2 years after completion of radiation.^14^

Although the concept of compliance differed, all evaluations could be performed either subjectively or objectively. The subjective evaluation method was widely used because of its advantages, such as being equipment free and low cost. However, this method is limited by inevitable subjectivity. On the patient side, beyond the Hawthorne effect, the outcomes of patients’ self-reported compliance may be affected by forgetting to record exercise, lacking time to record, fatigue, or app/internet issues (if patients must log exercise dates online).^20^ On the researcher side, a lack of quantitative criteria for performance levels also inevitably results in subjectivity in judgment.

In contrast, the objective evaluation method avoids the shortcomings of subjectivity to some extent. Nevertheless, the extensive application of objective methods is restricted by issues such as the need for specific equipment, higher costs, and technological difficulties.^20^ Moreover, the causes of noncompliance cannot be easily revealed by recording only the percentage of the prescribed exercises that were performed. Experts have recommended that combined methods including subjective and objective evaluations should be used to multidimensionally assess relevant information and obtain the most accurate compliance data.^37^ From the analyses, introducing factors regarding performance levels and the reason for noncompliance into the equipment design appears promising.

### Sociodemographic, illness-associated, and treatment-associated factors should be emphasized

According to previous studies, whether the sex and age of patients with HNC affect exercise compliance was unclear,^10^^,^^19^^,^^24^, ^25^, ^26^^,^^31^^,^^32^ possibly because of differences in target populations, as well as sample selection bias. Therefore, whether and how sex and age affect exercise compliance must be further investigated. Notably, approximately 60% of patients with HNC were ≥ 60 years of age at the time of diagnosis.^38^ Compared with younger patients, older patients may have more difficulties in learning exercise skills because of diminished cognitive ability.^39^ Furthermore, older patients may be more likely to feel exhausted, fatigued, or uncomfortable during exercise because of diminished physical function, thus decreasing their compliance with the required exercises.^39^ Therefore, targeted training and more flexible exercise plans are necessary, particularly for older people with poor endurance. Another factor associated with poor outcomes of swallowing exercise is smoking, which can cause poorer response to radiotherapy and increase radiotherapy-associated toxicity.^40^ Consequently, this factor should also be assessed.

After discharge, most economically disadvantaged patients living in rural areas in developing countries may not pay attention to their symptoms because of cost implications and/or living far from rehabilitation centers.^41^ As shown in a study led by Chen, the compliance rate decreased gradually with decreasing income.^31^ This finding is understandable, given that the economic burden of patients with low incomes may make them worry more about their family's economic status than their own quality of life, thus decreasing their motivation to comply with rehabilitation exercises. However, different countries may have different social security and medical care systems,^42^ and differences in whether/how the cost of rehabilitation is covered might directly affect compliance with swallowing exercises among patients with HNC, and therefore, rehabilitation outcomes.

Increased and uncontrolled pain and radiotherapy-associated toxicity may also impair patients' ability to complete swallowing exercises, particularly their exercise tolerance.^13^^,^^14^^,^^21^ Similar results have also been shown in another study.^43^ A study led by Starmer has reported that patients who received gabapentin (a painkiller) in the first week of radiotherapy, compared with patients who did not treat pain with gabapentin, showed better outcomes in pain relief and maintaining swallowing function.^44^ Hence, the management of pain and toxicity should be further investigated.

### Intervention strategies and perspectives

#### Multidisciplinary intervention is becoming a standard model for the management of swallowing exercises in patients with HNC

In previous studies, the interventions were delivered mainly by SLPs, SLTs, and speech therapists.^13^^,^^16^^,^^18^^,^^20^, ^21^, ^22^ Generally, both SLPs and SLTs are trained in anatomy, physiology, neurology, linguistics, phonetics, normal and pathological speech, language, voice, and swallowing.^45^^,^^46^ In contrast, SLPs focus on providing rehabilitation services to patients with varying neurological, oncological, or other disease processes that affect communication, cognition, and/or swallowing abilities.^47^ The roles of SLTs are aimed at the correction of speech problems for both elocution and medical disorders, including aphasia, motor speech disorders, HNC, voice disorders, and dysphagia.^45^ In contrast, speech therapists are trained to provide behavioral interventions for dysphonia to optimize patients' interaction with their environment.^48^^,^^49^ However, in practice, because SLPs, SLTs, and speech therapists have similar expertise, they all may be involved in the treatment of swallowing disorders.

Most published swallowing exercise protocols in patients with HNC have required intensive therapeutic services, thus placing a high demand on rehabilitation resources.^50^^,^^51^ However, owing to limited resources, the overall utilization rate was only 20.7% for SLPs and 26.2% for occupational/physical therapy services.^52^ Moreover, although unmet rehabilitation needs have been identified in 60%–70% of patients with HNC, professional rehabilitation therapists were reported to be reluctant to refer patients with HNC to general community-based services because of the uncertain quality of service.^53^^,^^54^ In developing countries, most swallowing rehabilitation treatments are provided by rehabilitation therapists. However, most of these rehabilitation therapists are transferred from other medical specialties after short-term training^55^; therefore, they often have insufficient professional knowledge and skills. Simultaneously, the work to improve compliance with swallowing exercises in patients with HNC is typically undertaken by nurses, whose professional qualifications should also be considered.

The issues of rehabilitation resources have highly limited the management of swallowing rehabilitation of patients with HNC. In recent years, multidisciplinary team–led interventions have been established in many groups and have been considered a standard model for the management of patients with HNC.^56^ Indeed, this multidisciplinary practice allows for rational distribution of the duties of health care providers and provides an effective means of optimizing health care resources for improved swallowing exercise results.

#### Refining the intervention procedure

As shown in the selected studies, although demographic screening and disease evaluation–exercise training–supervision and encouragement was the most common intervention procedure for increasing compliance with swallowing exercises, detailed investigations of the validity of the individual steps were not performed. For example, the time points of swallowing screening and evaluations, the exercise training content, and the order of provision of different training content were ambiguous, in agreement with the results reported by Ashley.^57^ In a study led by He, the PDCA cycle model was proposed to standardize education procedures, and the compliance with mouth opening exercise among patients with nasopharyngeal cancer has been effectively improved through this procedure.^32^ Given the clear advantages of this detailed procedure, we suggest that more evidence-based individual steps of intervention procedures should be developed.

#### Patients’ exercise tolerance and social support are important for completing swallowing exercises

The motivation of patients with HNC to comply with swallowing exercises is affected by their capacity and available social support.^58^ Long-term exercises often change patients' lifestyles; therefore, individuals’ beliefs are important in facilitating their adherence to these time-consuming rehabilitation exercises.^59^ Correspondingly, to promote cognition and beliefs regarding swallowing exercises among patients with HNC, different types of interventions were implemented in different forms at different stages of exercise, such as notification reminders, educational programs, and remote monitoring. However, the extent to which these social supports promote patient compliance, particularly among patients with poor individual capacity, such as poor exercise tolerance or passive attitude, should be further investigated.^22^

#### Further exploration of the availability of innovative equipment

Owing to limited clinical resources, most rehabilitation exercises were offered as home programs rather than in clinical settings.^16^ In these circumstances, m-Health systems have made enabled health care providers to conveniently remotely monitor exercise data, answer questions, and adjust training schedules for individuals.^10^^,^^16^^,^^20^ Moreover, the use of the m-Health systems has been reported to be cost-effective, thus providing patients with HNC with greater support in home-swallowing exercise while minimizing the burden of health service costs (eg., service time, consumables, and therapy resources) and patient-attributable costs (eg., travel).^60^ Importantly, biofeedback in apps provides a substantial advantage in making patients feel rewarded after even small improvements during swallowing training.^22^ This aspect is crucial to motivate patients to perform the required exercises.

Although a high overall satisfaction with m-Health Apps had been reported, this result might be affected by selection bias because patients with higher motivation typically volunteer to participate in telepractice, and the exercise motivation of patients might also be influenced by the use of a novel m-Health solution.^16^ In using m-Health apps, the notifications function of the app, which were used for reminding patients to exercise, were sometimes too frequent for patients with HNC, and internet issues have also become factors in participant dropout.^20^ Solutions designed to solve these problems by addressing these factors will improve the acceptance and motivation of patients to comply with swallowing exercise.

#### Applying systematic theory frameworks to improve intervention efficiency

According to our analysis, although behavioral change strategies had been used to unconsciously improve swallowing exercise compliance among patients with HNC, the poor descriptions of the strategies in most previous studies have greatly limited their generalization in clinical settings.^61^^,^^62^ Behavior change technology theory includes detailed strategies for goal setting, problem-solving, action plans, and feedback on behavior and thus might provide a potential solution. The interventions can be implemented effectively according to the content of BCTs.^63^ Pender's Health Promotion Model theory also had an advantage in identifying effective factors or barriers to swallowing exercise and allowed health care providers to provide targeted interventions for exercise compliance improvement.^27^ Furthermore, Orem self-care theory, which typically focuses on the self-care deficit of patients with HNC, may help clinicians design specific exercise plans according to patients' self-care abilities in different stages of HNC. This theory was particularly important for discharged patients.^29^

### Limitations

In this review, we aimed to identify specific factors and effective strategies for improving compliance with swallowing exercises in patients with HNC. Although different types of HNC have been examined in previous studies, such as the oral cavity, oropharynx, nasopharynx, larynx, hypopharynx, parotic gland, and parotic gland cancers, owing to the limited number of references, the effects of different tumor sites in exercise compliance could not be deduced. In addition, direct comparison of different findings was constrained by the differences in the definitions of compliance and exercise regime.

## Conclusions

To our knowledge, this is the first attempt to systematically analyze compliance with swallowing exercises among patients with HNC. According to the analysis, multiple factors affect compliance with swallowing exercises in patients with HNC, including sociodemographic factors, illness-associated factors, treatment-associated factors, and psychosocial factors. However, current interventions have focused mainly on psychosocial issues, such as developing various education programs; therefore, the other factors affecting swallowing exercise compliance should be further emphasized. Although the development of multidisciplinary teams and the application of innovative equipment have relieved the pressures on health care resources and economic status of patients to some extent, the professional qualifications of health providers and the availability of innovative equipment should be considered. In addition, measures including refining the intervention procedure and applying systematic theory frameworks should be performed to achieve better outcomes of compliance interventions.

## CRediT author statement

T.W. and R.D.: conceptualization and methodology. T.W. and C.C.: data curation. J.Z.: writing the original draft. H.Z., M.Z., and M.S.: literature search and data analysis. J.Z., X.W., T.W., S.C., and R.D.: reviewing and editing the article. All authors had full access to all the data in the study, and the corresponding authors had final responsibility for the decision to submit for publication. The corresponding authors attest that all listed authors meet authorship criteria and that no others meeting the criteria have been omitted.

## Declaration of competing interest

The authors declare no conflict of interest.

## Ethics statement

This study was approved by the Ethics Committee in the College of Nursing and Health of Zhengzhou University (IRB No. ZZUIRB-2022-85).

## Funding

This work was supported by the 10.13039/501100001809National Natural Science Foundation of China (Grant No. 82101505) and 10.13039/501100002858China Postdoctoral Science Foundation in 2018 (Grant No. 2018M630839).

## Data availability statement

The data that support the findings of our study are available upon reasonable request from the corresponding author, T.W.
