# Supplementary material for: Improving compliance with swallowing exercise to decrease radiotherapy-related dysphagia in patients with head and neck cancer
Source: Asia Pac J Oncol Nurs. 2022 Nov 19;10(1):100169. doi: 10.1016/j.apjon.2022.100169 (PMC9792737; doi:10.1016/j.apjon.2022.100169)
Supplement: Multimedia component 1 [file mmc1.docx]

**Appendix**

**Pubmed (Search on Aug 24, 2022)**

| Sets | Search strategies | Results |
| --- | --- | --- |
| #1 | “Head and neck Neoplasms”[MeSH Terms] OR “nasopharyngeal carcinoma”[MeSH Terms] OR "Cancer of Head and Neck*"[Title/Abstract] OR "head and neck cancer*"[Title/Abstract] OR "HNC*"[Title/Abstract] OR “head and neck tumor*”[Title/Abstract] OR “head and neck carcinoma *”[Title/Abstract] OR “head and neck cancers*”[Title/Abstract] OR “head & neck cancer*”[Title/Abstract] OR “head and neck neoplasm*”[Title/Abstract] OR “head and neck carcinoma*”[Title/Abstract] OR “head and neck tumors*”[Title/Abstract] OR “head and neck squamous cell carcinoma*”[Title/Abstract] OR “nasopharyngeal cancer *”[Title/Abstract] OR “nasopharyngeal neoplasm*”[Title/Abstract] OR “nasopharyngeal neoplasms*”[Title/Abstract] OR “nasopharyngeal*”[Title/Abstract] OR “nasopharynx cancer*”[Title/Abstract] OR “nasopharynx carcinoma*”[Title/Abstract] OR “nasopharyngeal carcinomas*”[Title/Abstract] OR “head and neck squamous cell carcinoma*”[Title/Abstract] OR“ pharyngeal cancers*”[Title/Abstract] OR “Pharyngeal cancers*”[Title/Abstract] | 374,043 |
| #2 | "Exercise Therapy"[MeSH Terms] OR "[Rehabilitation](https://www.ncbi.nlm.nih.gov/mesh/68012046)"[MeSH Terms] OR "Self-management"[MeSH Terms] OR "swallow training*"[Title/Abstract] OR "swallowing exercise*"[Title/Abstract] OR "deglutition training*"[Title/Abstract] OR "rehabilitation program*"[Title/Abstract] OR "rehabilitation plan*"[Title/Abstract] OR "rehabilitation intervention*"[Title/Abstract] OR "rehabilitative intervention*"[Title/Abstract] OR "rehabilitative interventions*"[Title/Abstract] OR "training programme"[Title/Abstract] OR "training plan*"[Title/Abstract] OR "training schedule*"[Title/Abstract] OR "exercise plan*"[Title/Abstract] OR "swallow therapy*"[Title/Abstract] OR "swallow intervention*"[Title/Abstract] OR "swallow treatment*"[Title/Abstract] OR “dysphagia exercise*”[Title/Abstract] OR "prophylactic exercises*"[Title/Abstract] OR "swallow preservation*"[Title/Abstract] OR “swallowing function improvement*”[Title/Abstract] OR "swallow therapy*"[Title/Abstract] OR "swallow intervention*"[Title/Abstract] OR "swallow treatment*"[Title/Abstract] OR “dysphagia exercise*”[Title/Abstract] OR "prophylactic exercises*"[Title/Abstract] OR "swallowing exercises *"[Title/Abstract] OR "swallowing intervention*"[Title/Abstract] OR "swallow treatment*"[Title/Abstract] OR “dysphagia exercise*”[Title/Abstract] OR "prophylactic exercises*"[Title/Abstract] OR “Preventive Rehabilitation*”[Title/Abstract] OR “ physical activity*”[Title/Abstract] OR "physical therapies*"[Title/Abstract] OR “exercise program*”[Title/Abstract] OR "physical therapies*"[Title/Abstract] OR "Prehabilitation*"[Title/Abstract] | 493,875 |
| #3 | "Patient Compliance"[MeSH Terms] OR “adherence*”[Title/Abstract] OR "Compliance*"[Title/Abstract] OR “Patient Adherence*”[Title/Abstract] OR “Patient Non Compliance*”[Title/Abstract] OR "implementation rates*"[Title/Abstract] OR “execution rate*”[Title/Abstract] OR “Patient engagement*”[Title/Abstract] OR “Patient Noncompliance*”[Title/Abstract] OR "Noncompliance, Patient*"[Title/Abstract] OR “Patient Adherence*”[Title/Abstract] OR "Patient Nonadherence*"[Title/Abstract] OR “Nonadherence, Patient*”[Title/Abstract] OR “Non-Adherent Patient*”[Title/Abstract] OR "Non Adherent Patient*"[Title/Abstract] OR “Patient NonCompliance*”[Title/Abstract] OR "Patient, Non-Adherent*"[Title/Abstract] OR “Patient Non-Adherence*”[Title/Abstract] | 311,836 |
| #4 | #1 AND #2 AND #3 | 110 |

**Embase (Search on Aug 24, 2022)**

| Sets | Search strategies | Results |
| --- | --- | --- |
| #1 | ('head'/exp OR head) AND ('neck'/exp OR neck) AND ('neoplasms'/exp OR neoplasms) OR ((((((((((((((((((cancer AND of AND head AND neck OR head) AND neck AND cancer OR HNC OR head) AND neck AND tumor OR head) AND neck AND carcinoma OR head) AND neck AND cancers OR head) AND & AND neck AND cancer OR head) AND neck AND neoplasm OR head) AND neck AND carcinoma OR head) AND neck AND tumors OR head) AND neck AND squamous AND cell AND carcinoma OR nasopharyngeal) AND cancer OR nasopharyngeal) AND neoplasm OR nasopharyngeal) AND neoplasms OR nasopharyngeal OR nasopharynx) AND cancer OR nasopharynx) AND carcinoma OR nasopharyngeal) AND carcinomas OR head) AND neck AND squamous AND cell AND carcinoma OR pharyngeal) AND cancers OR pharyngeal) AND cancers OR nasopharyngeal AND carcinoma:ti,ad,kw | 222,719 |
| #2 | 'kinesiotherapy'/exp or 'rehabilitation'/exp OR (((((((((((((((((((((((((((((((((((((((((((exercise AND therapies OR remedial) AND exercise OR rehabilitation) AND exercise OR rehabilitation) AND exercises OR exercise,) AND remedial OR therapies,) AND exercise OR therapy,) AND exercise OR remedial) AND exercises OR exercises,) AND rehabilitation OR exercise,) AND rehabilitation OR swallow) AND training OR swallowing) AND exercise OR deglutition) AND training OR rehabilitation) AND program OR rehabilitation) AND plan OR rehabilitation) AND intervention OR rehabilitative) AND intervention OR rehabilitative) AND interventions OR training) AND programme OR training) AND plan OR training) AND schedule OR exercise) AND plan OR swallow) AND therapy OR swallow) AND intervention OR swallow) AND treatment OR dysphagia) AND exercise OR prophylactic) AND exercises OR swallow) AND preservation OR swallowing) AND function AND improvement OR swallow) AND therapy OR swallow) AND intervention OR swallow) AND treatment OR dysphagia) AND exercise OR prophylactic) AND exercises OR swallowing) AND exercises OR swallowing) AND intervention OR swallow) AND treatment OR dysphagia) AND exercise OR prophylactic) AND exercises OR preventive) AND rehabilitation OR physical) AND activity OR physical) AND therapies OR exercise) AND program OR physical) AND therapies OR prehabilitation:ti,ad,kw | 761,184 |
| #3 | **'**patient compliance'/exp OR (((((((((((((((((((((((((((((((compliance, AND patient OR patient) AND adherence OR adherence OR patient) AND cooperation OR cooperation,) AND patient OR client) AND compliance OR client) AND compliances OR compliance,)  AND client OR client) AND adherence OR adherence,) AND client OR treatment) AND compliance OR compliance,) AND treatment OR treatment) AND compliances OR therapeutic) AND compliance OR compliance,) AND therapeutic OR therapeutic) AND compliances OR patient) AND 'non compliance' OR 'non compliance,') AND patient OR patient) AND non AND compliance OR patient) AND noncompliance OR noncompliance,) AND patient OR patient) AND nonadherence OR nonadherence,) AND patient OR 'non adherent') AND patient OR non) AND adherent AND patient OR 'non adherent') AND patients OR patient,) AND 'non adherent' OR patient) AND 'non adherence' OR 'non adherence,') AND patient OR patient) AND non AND adherence OR implementation) AND rates OR execution) AND rate OR patient) AND engagement:ab,kw,ti | 190,019 |
| #4 | #1 AND #2 AND #3 | 68 |

**Cochrane Library (Search on Aug 24, 2022)**

| Sets | Search strategies | Results |
| --- | --- | --- |
| #1 | MeSH descriptor: [Head and Neck Neoplasms] explode all trees | 6,647 |
| #2 | MeSH descriptor: [Nasopharyngeal Carcinoma] explode all trees | 241 |
| #3 | (Cancer of Head and Neck OR head and neck cancer OR HNC OR head and neck tumor OR head and neck carcinoma OR head and neck cancers OR head & neck cancer OR head and neck neoplasm OR head and neck carcinoma OR head and neck tumors OR head and neck squamous cell carcinoma OR nasopharyngeal cancer OR nasopharyngeal neoplasm OR nasopharyngeal neoplasms OR nasopharyngeal OR nasopharynx cancer OR nasopharynx carcinoma OR nasopharyngeal carcinomas OR head and neck squamous cell carcinoma OR pharyngeal cancers OR Pharyngeal cancers):ti,ab,kw | 14,013 |
| #4 | #1 OR #2 OR #3 | 17,407 |
| #5 | MeSH descriptor: [Exercise Therapy] explode all trees | 28,634 |
| #6 | MeSH descriptor: [Rehabilitation] explode all trees | 41,044 |
| #7 | Exercise Therapies OR Remedial Exercise OR Rehabilitation Exercise OR Rehabilitation Exercises OR Exercise, Remedial OR Therapies, Exercise OR Therapy, Exercise OR Remedial Exercises OR Exercises, Rehabilitation OR Exercise, Rehabilitation OR swallow training OR swallowing exercise OR deglutition training OR rehabilitation program OR rehabilitation plan OR rehabilitation intervention OR rehabilitative intervention OR rehabilitative interventions OR training programme OR training plan OR training schedule OR exercise plan OR swallow therapy OR swallow intervention OR swallow treatment OR dysphagia exercise OR prophylactic exercises OR swallow preservation OR swallowing function improvement OR swallow therapy OR swallow intervention OR swallow treatment OR dysphagia exercise OR prophylactic exercises OR swallowing exercises OR swallowing intervention OR swallow treatment OR dysphagia exercise OR prophylactic exercises OR Preventive Rehabilitation OR physical activity OR physical therapies OR exercise program OR physical therapies OR Prehabilitation ):ti,ab,kw | 165,954 |
| #8 | #5 OR #6 OR #7 | 194,465 |
| #9 | MeSH descriptor: [Patient Compliance] explode all trees | 126,96 |
| #10 | (Compliance, Patient OR Patient Adherence OR Adherence OR Patient Cooperation OR Cooperation, Patient OR Client Compliance OR Client Compliances OR Compliance, Client OR Client Adherence OR Adherence, Client OR Treatment Compliance OR Compliance, Treatment OR Treatment Compliances OR Therapeutic Compliance OR Compliance, Therapeutic OR Therapeutic Compliances OR Patient Non-Compliance OR Non-Compliance, Patient OR Patient Non Compliance OR Patient Noncompliance OR Noncompliance, Patient OR Patient Nonadherence OR Nonadherence, Patient OR Non-Adherent Patient OR Non Adherent Patient OR Non-Adherent Patients OR Patient, Non-Adherent OR Patient Non-Adherence OR Non-Adherence, Patient OR Patient Non Adherence OR implementation rates OR execution rate OR patient engagement):ti,ab,kw | 67,303 |
| #11 | #9 OR #10 | 743,28 |
| #12 | #4 AND #8 AND #11 | 204 |

**CINAHL (Search on Aug 24, 2022)**

| Sets | Search strategies | Results |
| --- | --- | --- |
| S1 | ( MH "Head and Neck Neoplasms+" OR MH “Nasopharyngeal Carcinoma+” ) OR TI ( Cancer of Head and Neck OR head and neck cancer OR HNC OR head and neck tumor OR head and neck carcinoma OR head and neck cancers OR head & neck cancer OR head and neck neoplasm OR head and neck carcinoma OR head and neck tumors OR head and neck squamous cell carcinoma OR nasopharyngeal cancer OR nasopharyngeal neoplasm OR nasopharyngeal neoplasms OR nasopharyngeal OR nasopharynx cancer OR nasopharynx carcinoma OR nasopharyngeal carcinomas OR head and neck squamous cell carcinoma OR pharyngeal cancers OR Pharyngeal cancers ) OR AB ( Cancer of Head and Neck OR head and neck cancer OR HNC OR head and neck tumor OR head and neck carcinoma OR head and neck cancers OR head & neck cancer OR head and neck neoplasm OR head and neck carcinoma OR head and neck tumors OR head and neck squamous cell carcinoma OR nasopharyngeal cancer OR nasopharyngeal neoplasm OR nasopharyngeal neoplasms OR nasopharyngeal OR nasopharynx cancer OR nasopharynx carcinoma OR nasopharyngeal carcinomas OR head and neck squamous cell carcinoma OR pharyngeal cancers OR Pharyngeal cancers ) | 215,718 |
| S2 | ( （MH "Exercise Therapy+" OR MH "Rehabilitation+") ) OR TI ( Exercise Therapies OR Remedial Exercise OR Rehabilitation Exercise OR Rehabilitation Exercises OR Exercise, Remedial OR Therapies, Exercise OR Therapy, Exercise OR Remedial Exercises OR Exercises, Rehabilitation OR Exercise, Rehabilitation OR swallow training OR swallowing exercise OR deglutition training OR rehabilitation program OR rehabilitation plan OR rehabilitation intervention OR rehabilitative intervention OR rehabilitative interventions OR training programme OR training plan OR training schedule OR exercise plan OR swallow therapy OR swallow intervention OR swallow treatment OR dysphagia exercise OR prophylactic exercises OR swallow preservation OR swallowing function improvement OR swallow therapy OR swallow intervention OR swallow treatment OR dysphagia exercise OR prophylactic exercises OR swallowing exercises OR swallowing intervention OR swallow treatment OR dysphagia exercise OR prophylactic exercises OR Preventive Rehabilitation OR physical activity OR physical therapies OR exercise program OR physical therapies OR Prehabilitation ) OR AB ( Exercise Therapies OR Remedial Exercise OR Rehabilitation Exercise OR Rehabilitation Exercises OR Exercise, Remedial OR Therapies, Exercise OR Therapy, Exercise OR Remedial Exercises OR Exercises, Rehabilitation OR Exercise, Rehabilitation OR swallow training OR swallowing exercise OR deglutition training OR rehabilitation program OR rehabilitation plan OR rehabilitation intervention OR rehabilitative intervention OR rehabilitative interventions OR training programme OR training plan OR training schedule OR exercise plan OR swallow therapy OR swallow intervention OR swallow treatment OR dysphagia exercise OR prophylactic exercises OR swallow preservation OR swallowing function improvement OR swallow therapy OR swallow intervention OR swallow treatment OR dysphagia exercise OR prophylactic exercises OR swallowing exercises OR swallowing intervention OR swallow treatment OR dysphagia exercise OR prophylactic exercises OR Preventive Rehabilitation OR physical activity OR physical therapies OR exercise program OR physical therapies OR Prehabilitation ) | 2,271,508 |
| S3 | MH "Patient Compliance+" OR TI ( Compliance, Patient OR Patient Adherence OR Adherence OR Patient Cooperation OR Cooperation, Patient OR Client Compliance OR Client Compliances OR Compliance, Client OR Client Adherence OR Adherence, Client OR Treatment Compliance OR Compliance, Treatment OR Treatment Compliances OR Therapeutic Compliance OR Compliance, Therapeutic OR Therapeutic Compliances OR Patient Non-Compliance OR Non-Compliance, Patient OR Patient Non Compliance OR Patient Noncompliance OR Noncompliance, Patient OR Patient Nonadherence OR Nonadherence, Patient OR Non-Adherent Patient OR Non Adherent Patient OR Non-Adherent Patients OR Patient, Non-Adherent OR Patient Non-Adherence OR Non-Adherence, Patient OR Patient Non Adherence OR implementation rates OR execution rate OR patient engagement) ) OR AB ( Compliance, Patient OR Patient Adherence OR Adherence OR Patient Cooperation OR Cooperation, Patient OR Client Compliance OR Client Compliances OR Compliance, Client OR Client Adherence OR Adherence, Client OR Treatment Compliance OR Compliance, Treatment OR Treatment Compliances OR Therapeutic Compliance OR Compliance, Therapeutic OR Therapeutic Compliances OR Patient Non-Compliance OR Non-Compliance, Patient OR Patient Non Compliance OR Patient Noncompliance OR Noncompliance, Patient OR Patient Nonadherence OR Nonadherence, Patient OR Non-Adherent Patient OR Non Adherent Patient OR Non-Adherent Patients OR Patient, Non-Adherent OR Patient Non-Adherence OR Non-Adherence, Patient OR Patient Non Adherence OR implementation rates OR execution rate OR patient engagement) ) | 856,363 |
| S4 | S1 AND S2 AND S3 | 296 |

**CNKI (Search on Aug 24, 2022)**

TKA=(头颈癌 + 头颈部肿瘤 + 鼻咽癌 ) AND TKA=(依从性 + 执行率+ 参与率 + 坚持) AND TKA= (吞咽训练 + 吞咽康复 + 吞咽干预 + 张口训练 +功能锻炼 + 康复锻炼 + 康复护理 + 管理 + 方案 + 模式)

Type = Journal(期刊论文), degree thesis/dissertation(学位论文)

Language = Chinese

RESULTS:295 hits

**Wan Fang Data (Search on Aug 24, 2022)**

(题名或关键词:(头颈癌 or 头颈部肿瘤 or 鼻咽癌 )) and (题名或关键词:(依从性 or 执行率or 参与率 or 坚持)) and (题名或关键词:(吞咽训练 or 吞咽康复 or 吞咽干预 or 张口训练 or功能锻炼 or 康复锻炼 or 康复护理 or 管理 or 方案 or 模式))

Type = Journal(期刊论文), degree thesis/dissertation(学位论文)

Language = Chinese

RESULTS:87 hits

**VIP (Search on Aug 24, 2022)**

M = (头颈癌 + 头颈部肿瘤 + 鼻咽癌) AND M = (依从性 + 执行率+ 参与率 + 坚持) AND M = (吞咽训练 + 吞咽康复 + 吞咽干预 + 张口训练 +功能锻炼 + 康复锻炼 + 康复护理 + 管理 + 方案 + 模式)

Limit the Subject to Medicine & Health

Type = Literature(文献)

Language = Chinese

RESULTS:48 hits
